# Psychometric properties of the persian version of the inventory of statements about self-injury (ISAS)

**DOI:** 10.1186/s40479-021-00168-4

**Published:** 2021-11-12

**Authors:** Omid Rezaei, Mojtaba Elhami Athar, Ali Ebrahimi, Elham Azamian Jazi, Sirwan Karimi, Shima Ataie, Ehsan Taheri, Mahboubeh Abbassian, Eric A Storch

**Affiliations:** 1 Psychosis Research Center, the University of Social Welfare and Rehabilitation Sciences, Tehran, Iran; 2 School of Behavioral Sciences and Mental Health (Tehran Institute of Psychiatry), Iran University of Medical Sciences, Tehran, Iran; 3 Candidate in Clinical Psychology, the University of Social Welfare and Rehabilitation Sciences, 1985713871 Tehran, Iran; 4 Segal Counseling & Psychological Services Center, Tehran, Iran; 5 University of Social Welfare and Rehabilitation Sciences, Tehran, Iran; 6 Department of Psychiatry and Behavioral Sciences, Baylor College of Medicine, TX Houston, USA

**Keywords:** Non-suicidal self-injury, Deliberate self-harm, Psychometric properties, Assessment, Persian version

## Abstract

**Background:**

Non-suicidal self-injury (NSSI) is a serious public health problem prevalent among adolescents and young adults. The present study examines the factor structure, internal consistency, and validity of the Persian version of the Inventory of Statements About Self-injury (ISAS), a self-report measure designed to comprehensively assess non-suicidal self-injury (NSSI).

**Methods:**

A total of 655 Iranian school-attending adolescents completed study measures online, and 246 youth (37.70 %) (M *age* = 15.38, *SD* ± 0.50; 53 % female) reported a history of NSSI at least once during their lifetime.

**Results:**

Confirmatory factor analysis supported the proposed two-factor model of ISAS (i.e., Interpersonal and Intrapersonal dimensions), which were internally consistent and yielded direct associations with converging correlates (e.g., depression, anxiety, irritability, and anger).

**Conclusions:**

Findings indicated that the Persian version of the ISAS has sound psychometric properties and is a valid and reliable self-report measure of NSSI.

## Background

Non-suicidal self-injury (NSSI) refers to any deliberate and direct destruction of body tissue in the absence of suicidal intention [1, 2]. NSSI manifests in various behavioral patterns such as cutting or carving the skin, burning the skin, or deliberately fracturing one’s bones [3] and is associated with psychiatric casenesses such as Borderline Personality Disorder (BPD), suicidality, anxiety, and depression e.g., [4–7]. The prevalence of NSSI is common with symptoms endorsed in approximately 20–30 % of adolescents in Turkey [8], Pakistan [9], Korea [3], Belgium [10], the USA [11], Germany [12], and Spain [13]. Concerning the prevalence of NSSI in Iran, a lifetime prevalence of 12.3 % without gender differences was reported among Iranian university students [14]; however, to our knowledge, no study has yet examined the prevalence of NSSI among Iranian school-attending adolescents.

NSSI often presents during adolescence and is a significant mental health challenge affecting about 70 % of children and adolescents with mental health problems e.g., [15–20]. Across six geographical regions (Asia, Australia/New Zealand, Canada, Europe, United Kingdom, USA) over 19 years, Swannell et al. (2014) reported that the prevalence of NSSI was 17.2 % for adolescents across different countries, schools, universities, and in community-based samples. Given the high prevalence of NSSI among adolescents [21, 22], assessing NSSI among this age group is of utmost importance.

The Inventory of Statements about Self-Injury (ISAS), developed by Klonsky et al. [4], assesses different NSSI functions. The ISAS consists of two parts. Part I assesses the frequency of 12 different types of NSSI behaviors, which were performed “intentionally and without suicidal intent,“ including banging/hitting, biting, burning, carving, cutting, wound picking, needle-sticking, pinching, hair pulling, rubbing skin against rough surfaces, severe scratching, and swallowing chemicals. Additionally, this part includes five further questions evaluating descriptive and contextual factors of NSSI, including the age of onset, the experience of pain during NSSI, whether NSSI is performed alone or around others, the time between the urge to self-injure and the act of NSSI, and if the person wants to end self-injuring or not. If participants confirm one or more NSSI behaviors, they are directed to complete Part II of the ISAS, which evaluates five intrapersonal and eight interpersonal NSSI functions (i.e., Affect Regulation, Anti-dissociation, Anti-suicide, Autonomy, Interpersonal boundaries, Interpersonal influence, Marking distress, Peer bonding, Self-care, Self-punishment, Revenge, Sensation seeking, and Toughness) through 39 items with three items for each function, rated on a 3‐point Likert type scale, ranging from 0 (*not relevant*) to 2 (*very relevant*). A higher score corresponds to a greater number of functions or motives for engaging in self-injury.

Given the importance of NSSI assessment, the ISAS has been translated and studied in various countries, including Sweden [23], Turkey [24], Australia [25], South Korea [3], Spain [26], Iran [27], Pakistan [9], and Norway [28]. In the original study, Klonsky et al. [4] examined the psychometrics of ISAS with 235 college students in the USA who had performed at least one NSSI behavior. Results of exploratory factor analysis (EFA) with Promax rotation indicated a robust two-factor solution. The first factor represented eight interpersonal functions (Autonomy, Interpersonal boundaries, Interpersonal influence, Peer-bonding, Revenge, Self‐care, Sensation‐seeking, and Toughness), and the second factor represented five Intrapersonal functions (Affect regulation, Anti-dissociation, Anti-suicide, Marking distress, and Self‐punishment). The same factor structure was replicated in Turkey [24], Australia [25], South Korea [3], and Pakistan [9]. Similarly, the two-factor model yielded a good fit with the sample of eating disorder or cluster B personality disorder patients in Spain [26]. In the most recent study with a sample of Norwegian students, the results confirmed the two-factor model of ISAS. The “*Marking distress*” function loaded on the interpersonal factor, which was loaded on the intrapersonal factor in the original study by Klonsky et al. [4]. The “self-care “function was also loaded on the intrapersonal factor, which belonged to the interpersonal factor in the original study [28]. In Iran, Zarghami et al. [27] examined the psychometrics of the ISAS among adult opioid and alcohol abusers. The EFA revealed a single-factor solution, which yielded an adequate fit in the subsequent confirmatory factor analysis (CFA). While important, Zarghami et al. [27] correlated seven error covariances in their one-factor solution, which may not provide a clear interpretation of the true factor structure [29, 30].

Beyond a stable factor structure, other psychometric properties of the ISAS are positive. The internal consistency of ISAS’ factors and the 13 functions were in the acceptable to excellent range in both community [3, 4, 24, 25, 28, 31] and clinical samples [9, 23, 26, 27]. Additionally, in support of their convergent validity, ISAS scores were associated with related constructs such as borderline personality symptoms, suicidality, depression, anxiety, impulsivity, and contextual variables such as the tendency to self-injure alone, suicidal ideations, and decreased resilience [3, 4, 24, 25, 27, 28, 31]; ISAS scores were also positively correlated with emotion dysregulation [26, 28] and negatively with distress tolerance [27].

While ISAS is a widely used measure to assess NSSI, its psychometrics have not been comprehensively examined in the Iranian youth sample, and thus, it is unclear if the findings from other cultures are generalizable to Iran [32–34]. For instance, in Iran, the predominant religious traditions strongly prohibit suicidal behavior. Moreover, this practice is evident in schools, where adolescents are taught that a suicide attempt is among Islam’s gravest sins, and if one attempts suicide, he/she will be deprived of paradise and its merits. Therefore, it is likely that individuals may feel guilty once they attempt suicide, and they may engage in NSSI instead of suicidal attempts, which may result in higher scores on the antisuicide function of NSSI. Thus, a separate study is needed to examine the ISAS in Iranian culture. Furthermore, despite the prevalence of NSSI among Iranian adolescents [35], NSSI is not a well-known and well-studied subject in Iran. Thus, this gap needs to be filled using valid NSSI measures. To this end, the current study investigated the factor structure, reliability, and validity of the ISAS with a sample of 655 Iranian school attending adolescents. CFAs were conducted to examine the proposed two-factor structure of the ISAS and the one-factor model, which has been proposed by Zarghami et al. [27] with an Iranian sample of adult opioid and alcohol abusers. Then, the reliability of the ISAS scores was estimated using Cronbach’s alpha coefficient (*α*) and mean inter-item correlations (MIC) values. Finally, the convergent validity of the ISAS scores was examined by calculating the associations between ISAS scores and related variables, such as depression and anxiety e.g., [24], suicidal ideation/ suicide attempts e.g., [3], and emotion regulation e.g., [26, 28]. We hypothesized that the two-factor model of the NSSI yield adequate model fit and enjoy acceptable internal consistency. We also expected ISAS scores to be correlated positively with external correlates of interest (e.g., depression, anxiety, irritability, and expressive suppression) e.g., [24, 26, 28].

## Methods

### Participants

Participants were 655 school-attending youth aged 13-17 years old who were recruited between November 2020 to April 2021. Of the 655 participants, 246 (*M*_*age*_ = 15.38, *SD* ± 0.50; 53 % female) respondents reported a history of NSSI at least once during their lifetime, and the analysis was computed based on the data from the latter group.

### Procedure

The ethics committee of the Psychosis Research Center, University of Social Welfare and Rehabilitation Sciences approved this study (code number: IR.USWR.REC.1399.223). Data were collected through a secured online platform. Therefore, we shared the online forms of the questionnaires with the social media groups of schools in Tehran, and 655 completed questionnaires were gathered.

## Measures

### ISAS

The ISAS [4] assesses the frequency and functions of NSSI and consists of two sections. The nature and psychometric properties have been reviewed previously.

*Persian ISAS*. For the present study, the ISAS was translated to Persian by two translators who were also fluent in English. Subsequently, Persian translations were translated back from Persian to English by a third, independent translator. Next, the back-translated English version of the ISAS was shared with a psychiatrist whose comments were implemented in reviewing and revising the measure.

### Emotion regulation questionnaire

The Emotion Regulation Questionnaire (ERQ; 36) is a 10-item self-report measure that includes two dimensions corresponding to two different emotion regulation strategies, i.e., cognitive reappraisal (6 items) and expressive suppression (4 items). Items are rated on a 7-point-Likert scale from 1 (*strongly disagree*) to 7 (*strongly agree*). The Persian version of the ERQ replicated the original two-factor model with adequate validity and excellent internal consistency for the expressive suppression (*α*: 0.85) and cognitive reappraisal (*α*: 0.87) dimensions [37]. Cronbach’s alpha and MICs for EQR dimensions can be retrieved from Table 1.

**Table 1:** Descriptive Statistics of ISAS, ERQ, and CCSM Variables (*n* = 247)

Measures	Mean (*SD*)	Skewness	Kurtosis	*α*	MIC
ISAS Interpersonal Functions	5.24 (6.45)	1.45	1.62	0.89	0.54
Interpersonal boundaries	0.77 (1.14)	1.49	1.50	0.60	0.35
Interpersonal influence	0.67 (1.09)	1.77	3.16	0.52	0.27
Toughness	1.07 (1.43)	1.34	1.22	0.75	0.49
Autonomy	0.87 (1.37)	1.64	2.02	0.79	0.56
Sensation seeking	0.74 (1.11)	1.56	1.85	0.52	0.29
Revenge	0.60 (1.08)	2.09	4.34	0.60	0.34
Peer-bonding	0.49 (1)	2.27	5.07	0.61	0.36
ISAS Intrapersonal Functions	7.54 (7.04)	0.87	0.10	0.87	0.54
Affect-regulation	1.77 (1.75)	0.67	-0.54	0.72	0.46
Self-punishment	1.25 (1.53)	1.24	0.98	0.67	0.41
Anti-dissociation	1.07 (1.37)	1.04	0.41	0.62	0.35
Anti-suicide	1.21 (1.71)	1.30	0.72	0.74	0.50
Self-care	1.25 (1.37)	1.04	0.42	0.56	0.32
Marking distress	0.94 (1.29)	1.26	0.72	0.79	0.56
ERQ					
Cognitive reappraisal	29.84 (6.08)	-0.56	1.28	0.62	0.22
Expressive suppression	17.23 (5.02)	-0.11	-0.39	0.57	0.25
CCSM					
Somatic symptoms	1.93 (1.95)	1.02	0.61	0.50	0.34
Sleep problems	1.37 (1.57)	0.64	-1.22	*	*
Inattention	1.23 (1.43)	0.80	-0.78	*	*
Depression	3.11 (2.65)	0.38	-1.10	0.48	0.31
Irritability	1.68 (1.50)	0.31	-1.32	*	*
Anger	1.76 (1.44)	0.28	-1.28	*	*
Mania	2.19 (2.22)	0.93	0.09	0.48	0.30
Anxiety	4.57 (3.68)	0.45	-0.90	0.78	0.55
Psychosis	1.35 (2.20)	1.70	2	0.75	0.60
Repetitive Thoughts & Behaviors	4.63 (4.02)	0.97	0.44	0.68	0.35
Substance use	7.81 (0.55)	-3.53	14.28	0.56	0.24
Suicidal Ideation/ Attempts	3.66 (0.63)	-1.70	1.613	0.59	0.42

*Note. ERQ*: Emotion Regulation Questionnaire; *CCSM*: DSM-5 Self-Rated Level 1 Cross-Cutting Symptom Measure—Child Age 11–17; *SD*: Standard deviation; *α*: Cronbach’s alpha coefficient; *MIC*: mean interitem correlation; *: *internal consistency was* *not measured because of having one item*

### DSM-5 self-rated level 1 cross-cutting symptom measure—child age 11–17

DSM-5 Self-Rated Level 1 Cross-Cutting Symptom Measure—Child Age 11–17 [1] contains 25 questions that assess 12 psychiatric domains, including depression, anger, irritability, mania, anxiety, somatic symptoms, inattention, suicidal ideation/attempt, psychosis, sleep disturbance, repetitive thoughts and behaviors, and substance use. Each item asks the child, age 11–17, to rate how much (or how often) he or she has been bothered by the specific symptom during the recent two weeks. Nineteen of the 25 items on the measure are rated on a 5-point scale ranging from 0 (*none or not at all*) to 4 (*severe or nearly every day*). The suicidal ideation, suicide attempt, and substance abuse items are rated on a “*Yes or No*” scale.

### Data analysis

Descriptive statistics of the study variables for the NSSI sample and the total sample are presented in Tables 1 and 2, respectively. We handled missing values using the series mean method in SPSS 20 and used the Boxplot method was to address outliers, resulting in a sample size of 655. Using data from 247 youth who had performed at least one NSSI behavior, we then performed CFAs (maximum likelihood estimator) using the Lisrel 8.80 software [38] to test the original two-factor [4] and the one-factor [27] models of the ISAS. Model fit indices included the comparative fit index (CFI) and the Tucker–Lewis index (TLI) ≥ 0.90 as indicating acceptable fit and the root mean square error of approximation (RMSEA) ≤ 0.08 indicating acceptable model fit [39, 40].

**Table 2:** Descriptive Statistics of ERQ and CCSM Variables (*n* = 655)

Measures	Mean (*SD*)	Skewness	Kurtosis	*α*	MIC
ERQ					
Cognitive reappraisal	29.66 (6.48)	-0.60	0.89	0.69	0.27
Expressive suppression	16.41 (5.51)	-0.12	-0.44	0.68	0.34
CCSM					
Somatic symptoms	1.42 (1.85)	1.47	1.77	0.58	0.41
Sleep problems	0.88 (1.38)	1.27	0.09	*	*
Inattention	0.85 (1.29)	1.33	0.47	*	*
Depression	2.17 (2.46)	0.94	-0.27	0.74	0.59
Irritability	1.19 (1.42)	0.86	-0.67	*	*
Anger	1.18 (1.34)	0.86	-0.51	*	*
Mania	1.67 (2.01)	1.17	0.67	0.44	0.29
Anxiety	2.96 (3.37)	1.08	0.09	0.80	0.58
Psychosis	0.76 (1.74)	2.66	6.76	0.77	0.62
Repetitive Thoughts & Behaviors	3.33 (3.68)	1.31	1.35	0.71	0.39
Substance use	7.89 (0.40)	-4.78	26.98	0.52	0.22
Suicidal Ideation/ Attempts	3.82 (0.45)	-2.71	6.70	0.50	0.33

*Note. ERQ*: Emotion Regulation Questionnaire; *CCSM*: DSM-5 Self-Rated Level 1 Cross-Cutting Symptom Measure—Child Age 11–17; *SD*: Standard deviation; *α*: Cronbach’s alpha coefficient; *MIC*: mean interitem correlation; *: *internal consistency was* *not measured because of having one item*

Next, we examined the internal consistency of the ISAS scores based on Cronbach’s alpha (*α*) and mean inter-item correlation (MIC) values. Αlpha coefficient ranges between 0 and 1, and since it tends to underestimate reliability when there are few items in a subscale, we calculated MIC values too, which are not dependent on the number of items in a scale and should be in the range of 0.15 to 0.50 to be considered adequate [41].

Finally, we evaluated the convergent validity of ISAS scores by examining Pearson correlation coefficients between the ISAS dimensions and correlates of interest (e.g., depression, anger, anxiety, suicidal ideation/ suicide attempts, and emotion regulation strategies).

## Results

Of the whole sample (*n* = 655), 247 (37.70 %) respondents reported a history of NSSI at least once during their lifetime. The most commonly endorsed means of NSSI were interfering with wound healing (69 %), carving (34 %), biting (28 %), pulling hair (24 %), banging or hitting (23 %), severe scratching (20 %), cutting (15 %), sticking self with needles (11 %), burning (11 %), rubbing skin against rough surfaces (11 %), pinching (6 %), and swallowing dangerous substances (3 %) (Table 3).

**Table 3:** Frequency of non-suicidal self-injury (*n*=247)

Behavior	Frequency					
	n	0-5	5-10	10-15	15-20	< 20
Cutting	36	23	5	5	2	1
Bitting	70	42	17	7	2	5
Burning	26	18	5	3	-	-
Carving	85	41	18	14	6	6
Pulling Hair	60	38	8	8	1	5
Severe Scratching	49	30	11	4	3	1
Banging or Hitting Self	58	31	12	8	4	3
Interfering w/wound healing (e.g., picking scabs)	171	84	27	38	8	14
Sticking Self w/ Needles	28	16	4	6	2	-
Swallowing Dangerous Substances	8	5	1	2	-	-
Rubbing Skin Against Rough Surface	27	15	4	5	2	1
Pinching	14	7	3	4	-	-
Others	-	-	-	-	-	-

### Confirmatory factor analysis

The results of confirmatory factor analysis showed that the one-factor (RMSEA = 0.101; CFI = 0.97, TLI = 0.96) and two-factor model of ISAS (RMSEA = 0.098; CFI = 0.97, TLI = 0.97) reached adequate fitness according to two fit indices. Nonetheless, we examined modification indices to improve the model fit. Accordingly, for the two-factor model, we added a path from the intrapersonal factor to the self-care function (Table 4). In the original study by Klonsky et al. [4], the self-care function was theoretically expected to load on the intrapersonal factor, but such a result was not found. Consequently, while the RMSEA value decreased, still, it was not in the acceptable recommended ≤ 0.08 range [39, 40] but was very close to it (RMSEA = 0.092; CFI = 0.98, TLI = 0.97). Nonetheless, some sources consider RMSEA ≤ 0.10 as adequate [42]. Thus, our modified two-factor model could also be considered adequate fit based on RMSEA ≤ 0.10 as recommended by Byrne [42], and because other fit indices (i.e., CFI and TLI) were in the excellent range (i.e., ≤ 0.95) while loadings were above the recommended threshold (< 40). Since the two-factor model has been supported in previous studies and since this model outperformed the one-factor model though slightly with respect to some fit indices (i.e., RMSEA and TLI), all the analysis from here onwards will be calculated based on the two-factor model.

**Table 4:** Factor loadings of ISAS functions (*n* = 247)

Function	Interpersonal Functions	Intrapersonal Functions
Affect-regulation		0.69
Self-punishment		0.64
Anti-dissociation		0.82
Marking distress		0.82
Self-care		0.80
Anti-suicide		0.66
Interpersonal boundaries	0.71	
Interpersonal influence	0.66	
Toughness	0.83	
Autonomy	0.81	
Sensation seeking	0.72	
Revenge	0.69	
Peer-bonding	0.68	

### Internal consistency and correlation between the ISAS scores

According to Cronbach’s alpha and MIC values, the internal consistency of the modified ISAS factors was good (Table 1). Concerning the ISAS 13 functions, the internal consistency ranged from 0.52 (Interpersonal influence and Sensation seeking) to 0.79 (Autonomy) for interpersonal functions and from 0.62 (Anti-dissociation) to 0.79 (Marking distress) for Intrapersonal functions based on Cronbach’s alpha, while all of the functions were in the acceptable range when relying on MIC values. A significant zero-order correlation was found between ISAS factor scores, which was: r ^Interpersonal−Intrapersonal^ = 0.79.

### Convergent validity

Both Interpersonal and Intrapersonal factors were positively related to sleep problems, inattention, depression, irritability, anger, mania, anxiety, psychosis, expressive suppression, and repetitive thoughts and behaviors, but negatively with substance use and suicidal ideation/attempts. Only the Intrapersonal factor had a significant positive relationship with somatic symptoms (Table 5).

**Table 5:** Correlations between ISAS scores and external correlates

Measures	ISAS Interpersonal Functions	ISAS Intrapersonal Functions
ISAS Interpersonal Functions	-	
ISAS Intrapersonal Functions	0.79^**^	-
**ERQ**		
Cognitive reappraisal	-0.002	-0.008
Expressive suppression	0.18^**^	0.22^**^
**CCSM**		
Somatic symptoms	0.05	0.13^**^
Sleep problems	0.16^**^	0.20^**^
Inattention	0.15^**^	0.24^**^
Depression	0.28^**^	0.33^**^
Irritability	0.33^**^	0.37^**^
Anger	0.31^**^	0.31^**^
Mania	0.29^**^	0.21^**^
Anxiety	0.40^**^	0.41^**^
Psychosis	0.37^**^	0.30^**^
Repetitive Thoughts & Behaviors	0.25^**^	0.28^**^
Substance use	-0.25^**^	-0.18^**^
Suicidal Ideation/ Attempts	-0.44^**^	-0.44^**^

*Note*. *ERQ*: Emotion Regulation Questionnaire; *CCSM*: DSM-5 Self-Rated Level 1 Cross-Cutting Symptom Measure—Child Age 11–17; **p < .001

## Discussion

The current study examined the psychometric properties and factor structure of ISAS with a sample of Iranian school-attending youth. Our results indicated that the two-factor model initially demonstrated a fair fit. As such, we used the modification indices to improve model fit; thus, the self-care function loaded on the intrapersonal dimension. We concluded that our modified two-factor model has an adequate fit because despite the RMSEA not reaching the recommended range ≤ 0.08, other fit indices were in the excellent range, and factor loadings were significantly higher than the threshold of 0.40. Our results concerning the RMSEA are consistent with previous research. For example, in a study in South Korea [3], the RMSEA was 0.10, while CFI was 0.91. Similarly, in the Turkish study [24], the results yielded an RMSEA of 0.08 and CFI of 0.97. Also, in line with previous studies [25, 28], our results indicated that self-care function aligned as an intrapersonal function. This was theoretically expected but not found in the original study by Klonsky et al. [4].

Echoing previous studies [3, 4, 24, 25, 28, 31], our results indicated that the internal consistency of the ISAS dimensions was good. In addition, all of the functions had acceptable MIC values and were internally consistent. These results are very consistent with the findings of Klonsky et al. [4].

The current study also examined associations between ISAS scores and external criterion measures to bolster what is known about the convergent validity of the Persian version of ISAS. Consistent with previous studies [4, 24, 26, 43–48], both ISAS dimensions were positively related to sleep problems, inattention, depression, irritability, anger, mania, anxiety, psychosis, and repetitive thoughts and behaviors; also, only Intrapersonal factor had a significant positive relationship with somatic symptoms. Our results also indicated that ISAS scores had significant positive correlations with expressive suppression, while they were not correlated with cognitive reappraisal. This finding is consistent with Gross and John [36] in that reappraisers experience and express greater positive and lesser negative emotions, whereas suppressors experience and express lesser positive emotions, yet experience greater negative ones. To our surprise, both Intrapersonal and Interpersonal dimensions were negatively associated with substance use and suicidal ideation/attempt. This might be due to the fact that our sample included 13-17 years old school attending adolescents who usually do not have access to illegal substances. Also, in Iran, suicidal behavior is strongly prohibited by religious and socio-cultural factors. For instance, in Iran’s schools, based on Islamic instruction, adolescents learn that suicide attempt is amongst gravest sins in Islam, and such an attempt, would deprive the individual of the paradise and its merits. Thus, individuals may feel guilty when they think about attempting suicide, and they may engage in NSSI instead of suicidal attempts. Notwithstanding, this aspect of the measure’s convergent validity remains unclear, and further studies are needed to explore this finding. In sum, our results support the convergent validity of the interpretation of the ISAS dimension in Iranian school-attending adolescents.

## Limitations

Our findings should be interpreted in the context of several limitations. First, we used only self-report measures. Therefore, correlations between ISAS scores and external correlates may partly be explained by shared method variance. Second, since the current study had a cross-sectional nature, conclusions about causality between ISAS scores and correlated variables should not be drawn. Finally, the study sample included only school attending adolescents, so future studies are recommended to study the psychometric of the ISAS with clinical samples.

## Conclusions

Overall, the Persian version of the ISAS can be widely used as a valid and reliable self-report measure of NSSI in research studies and clinical settings with adolescents in Iran as it yielded excellent internal consistency and associations with the external correlates of interest.

## Data Availability

The datasets generated during and/or analyzed during the current study are available from the corresponding author on reasonable request.
